# Gut microbiota on admission as predictive biomarker for acute necrotizing pancreatitis

**DOI:** 10.3389/fimmu.2022.988326

**Published:** 2022-08-29

**Authors:** Menglian Zou, Zihan Yang, Yue Fan, Liang Gong, Ziying Han, Li Ji, Xiaomin Hu, Dong Wu

**Affiliations:** ^1^ Department of Gastroenterology, State Key Laboratory of Complex Severe and Rare Diseases, Peking Union Medical College Hospital, Chinese Academy of Medical Sciences & Peking Union Medical College, Beijing, China; ^2^ Department of Medical Research Center, State Key Laboratory of Complex Severe and Rare Diseases, Peking Union Medical College Hospital, Chinese Academy of Medical Science & Peking Union Medical College, Beijing, China

**Keywords:** acute pancreatitis, necrotizing pancreatitis, gut microbiota, dysbiosis, prognosis

## Abstract

**Background:**

Acute necrotizing pancreatitis (NP), a severe form of acute pancreatitis (AP), has higher mortality and worse outcome than non-necrotizing pancreatitis (non-NP). Infected NP is a devastating subgroup of NP. To date neither NP nor infected NP has robust prediction strategies, which may delay early recognition and timely intervention. Recent studies revealed correlations between disturbed gut microbiota and AP severity. Some features of intestinal microbiota have the potential to become biomarkers for NP prediction.

**Methods:**

We performed 16S rRNA sequencing to analyze gut microbiota features in 20 healthy controls (HC), and 58 AP patients on hospital admission. The AP patients were later classified into NP and non-NP groups based on subsequent diagnostic imaging features. Random forest regression model and ROC curve were applied for NP and infected NP prediction. PIRCUSt2 was used for bacterial functional pathway prediction analysis.

**Results:**

We found that the three groups (HC, NP, and non-NP) had distinct microorganism composition. NP patients had reduced microbial diversity, higher abundance of *Enterobacteriales*, but lower abundance of *Clostridiales* and *Bacteroidales* compared with the non-NP group. Correlation analyses displayed that intestine bacterial taxonomic alterations were related to severity, ICU admission, and prognosis. By pathway prediction, species more abundant in NP patients had positive correlation with synthesis and degradation of ketone bodies, and benzoate degradation. *Enterococcus faecium* (ASV2) performed best in discriminating NP and non-NP patients. *Finegoldia magna* (ASV3) showed the maximal prediction capacity among all ASVs and had comparable accuracy with Balthazar CT to detect patients with infected NP.

**Conclusions:**

Our study suggests that NP patients have distinct intestinal microbiota on admission compared to non-NP patients. Dysbiosis of intestinal microbiota might influence NP progression through ketone body or benzoate metabolism. *Enterococcus faecium* and *Finegoldia magna* are potential predictors for NP and infected NP. Our findings explore biomarkers which may inform clinical decision-making in AP and shed light on further studies on NP pathophysiology and management.

## Introduction

Acute pancreatitis (AP) is a leading cause for acute admission due to gastrointestinal diseases and results in enormous morbidity and mortality (1, 2). The incidence of AP is around 34 per 100,000 cases worldwide, and the overall incidence increases by 3.07% per year, inducing substantial medical and social burden (3, 4).

There are two subtypes of AP: interstitial edematous pancreatitis and necrotizing pancreatitis. Necrotizing pancreatitis (NP), a non-mild AP manifestation, represents (peri-)pancreatic parenchymal necrosis and accounts for 10%-20% of AP patients (5, 6). Developing NP leads to unfavorable prognosis, that is, the mortality is about 15%, and for those with infected NP (INP), even as high as 30%-39% (7). Early recognition of patients who are prone to develop NP is crucial, particularly on clinical admission, because proper triage, close monitoring, early intervention, and multidisciplinary treatment may prevent organ dysfunction and lethal outcome (8). Timely aggressive antibiotic treatment and proper drainage or debridement is indicated in patients with infected NP. Therefore, early prediction of NP with or without infection is of clinical significance.

However, neither etiologic factors (gallstones, alcohol use, etc.) nor demographics features are conclusively risk factors to necrosis formation (9). A series of studies have evaluated the prediction potential of a variety of biomarkers and scoring systems for NP or infected NP, but none are robust enough. Some studies have investigated lab indicators for necrosis prediction, but none have been solid (10, 11). Existing scoring systems, for instance the Ranson score, the acute physiology and chronic health evaluation-II (APACHE-II), the computed tomography severity index (CTSI), or the Bedside Index of Severity in Acute Pancreatitis (BISAP) could not differentiate the aseptic or infected necrosis (12). Thus more efficient and cost-effective methods for prediction of NP and infected NP are needed.

Recent decades have witnessed that gut microbiota alteration were closely related to the progression of AP. Around 60% of patients with AP had varying degrees of intestinal barrier injury, which led to increased intestinal mucosal permeability and intestinal bacterial translocation, furthering the exacerbation of NP, presumably developing MODS in the end (13). Moreover, a previous study indicated that severe AP (SAP) had a lower relative abundance of beneficial bacteria (*Bifidobacterium* etc.), and a higher relative abundance of potentially pathogenic bacteria (*Enterococcus* etc.), in comparison with MAP patients (14). These studies inspired the potential role of the microbiota in NP progression. It has been evidenced that (peri-)pancreatic collections are liable to be infected with gut microorganisms (15). Thus, gut microbiome may help with pathogen prediction and antibiotic selection. Therefore, clarifying the role of dysbiosis in NP will not only enrich our knowledge regarding the “gut microbiota-pancreas axis”, but also shed light on suitable therapy.

To determine the prediction potential of gut microbiota for NP outcome on admission, we performed a prospective, observational study. We studied the association between intestinal microflora and the prognosis of patients with NP to unveil potential microbiota biomarkers and lay the foundation for future research translation. We hypothesized that key features of the gut microbiome (diversity, community composition, certain ASVs) would predict exacerbation of NP patients on admission.

## Materials and methods

### Study population and sample collection

We performed a prospective, observational study in a single center. We enrolled 20 healthy controls and 58 AP patients at Peking Union Medical College Hospital (PUMCH), Beijing, China. Healthy controls were community workers or students enrolled from PUMCH who were age and gender matched to the patient population. They were free of intestinal, metabolic, cardiovascular or cerebrovascular diseases. Female volunteers were not pregnant. They were with no known allergens. They were neither on any treatment interfering bowel function, nor on any specific diet for weight loss or certain goals. Patients were selected from our database by a stratified random sampling method. Patients diagnosed with AP based on the Revised Atlanta Classification were included (5). Enrolled patients must be admitted within 24 h after symptom onset. Exclusion criteria included cancer, chronic pancreatitis, gastroenteritis, inflammatory bowel disease (IBD), irritable bowel syndrome (IBS), necrotizing enterocolitis, and immunocompromised status. Patients on antibiotics, probiotics, Chinese herbs, or laxatives within two months before enrollment were also excluded. All enrolled patients provided informed consent. Our study was approved by the PUMCH Ethics Committee (Identifier: JS1826, Date of approval: 20^th^ February 2018. Period of validity: February 2018 to August 2020). All clinical characteristics were recorded according to standard procedures. Peripheral venous blood from the participants was tested on admission and through disease progression. We collected stool samples with rectal swabs from each participant on admission. Protocols of sample and data collection were described previously (16, 17).

### Demographic and clinical data

We went through medical records to extract clinical data, such as gender, age, weight, height, comorbidities, lab results, imaging studies, severity stratification, complications, and outcomes. AP was graded into mild AP (MAP), moderately severe AP (MSAP), and severe AP (SAP) (5). NP was defined as pancreatic and/or peripancreatic necrosis (5). Infected NP was either diagnosed based on a positive culture of fine needle aspiration (FNA) of pancreatic and/or peripancreatic necrosis, or the presence of gas in CECT, or the positive culture from the first drainage procedure under sterile conditions. The APACHE II (18), the Balthazar scores (19), and the Sequential Organ Failure Assessment (SOFA) scores (20), were used to evaluate disease severity when NP patients were distinguished from non-NP patients. Local and systematic complications were also defined according to past studies (5, 21). Local complications consisted of acute peripancreatic fluid collection (APFC), acute necrotic collection (ANC), walled-off necrosis (WON), pseudocyst, and infected necrosis. Systematic complications comprised systemic inflammatory response syndrome (SIRS), acute respiratory distress syndrome (ARDS), shock, acute kidney injury (AKI), altered mental status, myocardial injury, liver damage, abdominal compartment syndrome (ACS), and bowel obstruction. Mortality, organ failure, duration of hospital stay, and days of ICU stay were chosen as clinical outcomes.

### DNA collection and extraction, 16S rRNA gene sequencing and processing

Microbial DNA samples were extracted from rectal swab as previously described using the bead-beating method (22) and then went through gel electrophoresis to determine the quality of the DNA sample. DNA was diluted to 1 ng/L with sterile water and then served as templates to run PCR to amplify the V3-V4 regions (23). High-fidelity enzymes, Phusion^®^ High-Fidelity PCR Master Mix with GC Buffer (New England Biolabs, Ipswich, MA, USA), and primers with Barcode were employed for PCR. After establishing a sequencing library of 16S rRNA V3-V4 regions, an Illumina MiSeq platform (Illumina Inc., San Diego, CA, USA) was used to pool and sequence the purified amplicons.

The bioinformatic analyses of 16s amplicons were accomplished relying on the EasyAmplicon (Version 1.10) (24). Dereplication was achieved by VSEARCH (version 2.15) (25) and the sequences were then denoised into amplicon sequence variants (ASV) with the implemented command in USEARCH (Version 10.0) (26), and an ASV table was generated. The sintax algorithm of USEARCH recognized taxonomic classification of ASVs according to the Ribosomal Database Project (RDP) training set v16 (27).

### Sequencing data analysis and visualization

The R package vegan (v2.5-6) was applied on Alpha diversity analysis (28). Differences in richness index were evaluated by Turkey’s HSD test. The usearch achieved the weighted UniFrac distance matrix. PCoA (Principal coordinate analysis) was utilized to calculate beta diversity, and Adonis test was applied to verify the difference among groups. The R package ggplot2 visualized the alpha and beta diversity. A Venn diagram illustrating overlapping ASVs among the three groups was created by the R package VennDiagram.

The taxonomic composition was presented either as a stacked bar plot or a chord plot at different taxonomic levels by the ggplot2 package. As for between-group ASV comparisons, the EdgeR method was utilized to calculate the p-value and identify taxonomic features significantly different among groups, and FDR was calculated using the Benjamini-Hochberg method. The volcano plot and Manhattan plot exhibited differential ASVs among groups. Linear discriminant analysis effect size (LEfSe) (http://huttenhower.sph.harvard.edu/galaxy) was utilized for comparison of the intestinal microbiome composition.

### Feature selection by random forest model

Using 16s sequence profiles and clinical parameters, we randomly divided the samples into a training set and a testing set. We built up a random forest regression model to establish a training set for 70% of the samples and a testing set for the remaining 30% using the R package randomForest. Then, tenfold cross-validation method was applied to the training set. An optimal set of microbial variables was built at the lowest cross-validation error to predict the AP severity, as evaluated by the APACHE II index. We constructed the predictive model with the most important variables, which further went through ROC calculation to distinguish NP patients from non-NP. The pROC R package was used to calculate the confidence intervals for the ROC curves.

### Prediction of gut microbiota phenotype

The USEARCH-otutab command classified the sequences based on the Greengene database (29). Then, the sequence library was further processed by Bugbase for phenotype prediction of gut flora (30). As for pathway prediction analysis, we used PICRUSt2 to predict the possible functional composition based on GO and KEGG database (31). STAMP software (v2.1.3) was used for statistical comparison of the predicted pathways of the microbial community (Welch’s t-test).

## Results

### Clinical characteristics of enrolled patients

The study enrolled a total of 58 AP patients and 20 healthy controls. The AP patients were classified into NP and non-NP groups based on a careful evaluation on diagnostic imaging. **Table 1** demonstrated the demographic and clinical features of these two groups. The non-NP group had a lower CRP level (median 115.0, IQR 21.5-160.0 vs. median 160.0, IQR 131.8-232.1; *p* < 0.001) than the NP group. Several scoring systems including APACHE II, SOFA, and Balthazar score were significantly different (*p* < 0.001) between the groups. Furthermore, the NP group had a higher risk of SAP development (89.5% vs. 2.6%, *p* < 0.001), and certain systematic complications including SIRS (89.5% vs. 28.2%, *p* < 0.001), ARDS (73.7% vs. 17.9%, *p* < 0.001), AKI (42.1% vs. 10.3%, *p* = 0.005), shock (36.8% vs. 2.6%, *p* < 0.001), ACS (26.3% vs. 5.1%, *p* = 0.021), liver damage (42.1% vs. 5.1%, *p* = 0.001), myocardial injury (26.3% vs. 0.0%, *p* = 0.001), and sepsis (47.4% vs. 7.7%, *p* = 0.001) compared to the non-NP group. Besides, more NP patients were complicated with organ failure (84.2% vs. 20.5%, *p* < 0.001) and ICU admission (89.5% vs. 7.7%, *p* < 0.001). And NP patients had a longer organ failure duration (median 74.0, IQR 48.0-276.0 vs. median 0.0, IQR 0.0-0.0; *p* < 0.001), a longer ICU stay (median 7.0, IQR 5.0-10.5 vs. median 0.0, IQR 0.0-0.0; *p* < 0.001), and a longer hospital stay (median 23.0, IQR 17.5-30.0 vs. median 6.0, IQR 3.0-11.5; *p* < 0.001) compared with non-NP patients.

**Table 1:** Demographic and clinical characteristics of two groups.

Variables	NP (n = 19)	Non-NP (n = 39)	*p*-value
Age (years), mean (SD)	41.8 (11.4)	47.2 (15.8)	0.054
Male, n (%)	12 (63.2)	18 (46.2)	0.228
BMI (kg/m^2^), mean (SD)	26.1 (4.1)	26.2 (3.2)	0.723
Overweight (BMI 25–29.9 kg/m^2^), n (%)	11 (57.9)	19 (48.7)	0.515
Obesity (BMI ≥ 30 kg/m^2^), n (%)	1 (5.3)	4 (10.3)	0.528
Smoking, n (%)	7 (36.8)	9 (23.1)	0.275
Drinking, n (%)	6 (31.6)	9 (23.1)	0.491
Comorbid abnormalities, n (%)			
Hypertension	6 (31.6)	13 (33.3)	0.895
Diabetes	6 (31.6)	7 (17.9)	0.247
Fatty liver	14 (73.7)	26 (66.7)	0.591
Laboratory examinations			
Triglyceride (mmol/L), median (IQR)	13.6 (1.2, 18.7)	2.2 (1.2, 18.5)	0.456
CRP (mg/L), median (IQR)	160.0 (131.8, 232.1)	115.0 (21.5, 160.0)	<0.001
Etiology, n (%)			
Biliary	6 (31.6)	19 (48.7)	0.220
Hypertriglyceridemia	12 (63.2)	16 (41.0)	0.117
Alcohol consumption	1 (5.3)	4 (10.3)	0.528
APACHE II, median (IQR)	9.0 (7.0, 11.0)	3.0 (2.0, 5.5)	<0.001
SOFA score, median (IQR)	4.0 (2.5, 7.0)	1.0 (0.0, 1.0)	<0.001
Balthazar score E, n (%)	8 (42.1)	0 (0.0)	<0.001
Disease severity, n (%)			
MAP	0 (0.0)	20 (51.3)	<0.001
MSAP	2 (10.5)	18 (46.2)	0.008
SAP	17 (89.5)	1 (2.6)	<0.001
Local complications, n (%)			
APFC	19 (100.0)	15 (38.5)	<0.001
Pancreatic pseudocyst	1 (5.3)	3 (7.7)	0.734
ANC	14 (73.7)	0 (0.0)	<0.001
WON	2 (10.5)	0 (0.0)	0.041
Infected necrosis	7 (36.8)	0 (0.0)	<0.001
Systematic complication, n (%)			
SIRS	17 (89.5)	11 (28.2)	<0.001
ARDS	14 (73.7)	7 (17.9)	<0.001
AKI	8 (42.1)	4 (10.3)	0.005
Shock	7 (36.8)	1 (2.6)	<0.001
ACS	5 (26.3)	2 (5.1)	0.021
Liver damage	8 (42.1)	2 (5.1)	0.001
Myocardial injury	5 (26.3)	0 (0.0)	0.001
Altered mental status	1 (5.3)	0 (0.0)	0.152
Sepsis	9 (47.4)	3 (7.7)	0.001
Bowel obstruction	6 (31.6)	5 (12.8)	0.090
Outcome			
Organ failure, n (%)	16 (84.2)	8 (20.5)	<0.001
Organ failure duration (h), median (IQR)	74.0 (48.0, 276.0)	0.0 (0.0, 0.0)	<0.001
ICU, n (%)	17 (89.5)	3 (7.7)	<0.001
ICU stay (days), median (IQR)	7.0 (5.0, 10.5)	0.0 (0.0, 0.0)	<0.001
Hospital stay (days), median (IQR)	23.0 (17.5, 30.0)	6.0 (3.0, 11.5)	<0.001
Death, n (%)	1 (5.3)	0 (0.0)	0.152

NP, necrotizing pancreatitis; SD, standard deviation; BMI, body mass index; CRP, C-reactive protein; IQR, interquartile range; APACHE II, the Acute Physiology and Chronic Health Evaluation II score; SOFA, the Sequential Organ Failure Assessment score; MAP, mild acute pancreatitis; MSAP, moderately severe acute pancreatitis; SAP, severe acute pancreatitis; APFC, acute peripancreatic fluid collection; ANC, acute necrotic accumulation; WON, walled-off necrosis; ARDS, acute respiratory distress syndrome; AKI, acute kidney injury; ACS, abdominal compartment syndrome; SIRS, systemic inflammatory response syndrome; ICU, intensive care unit. CRP was tested at the time of NP diagnosis. APACHE II and SOFA were also calculated at the time of NP diagnosis.

### Microbial profile of patients diagnosed with NP and non-NP

**Figure 1:**
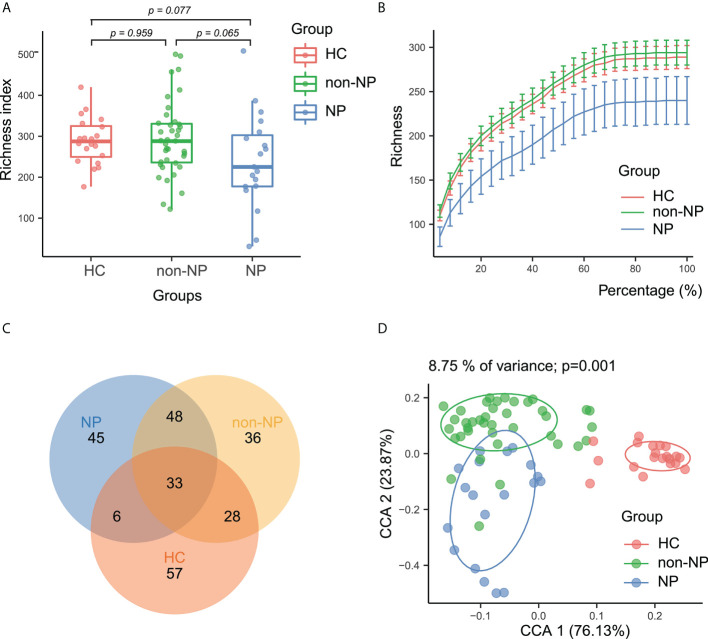
Alpha and beta diversity analysis between NP, non-NP and healthy control groups. **(A)** Alpha diversity based on richness index. Box-plot features represent the median (central line), upper and lower quartiles (box), and the maximum and minimum values of the data (bars). **(B)** A refraction curve demonstrating species richness. **(C)** A Venn diagram demonstrating the existence of ASVs in each group. **(D)** Beta diversity analysis based on CPCoA plot. Each symbol represents the gut microbiota of a sample. NP, necrotizing pancreatitis; non-NP, non-necrotizing pancreatitis; CPCoA, constrained principal coordinate analysis.

To determine whether the gut microbial composition may be predictive of NP and AP severity, we collected 58 rectal swabs from AP patients and 20 from healthy controls and performed 16S rRNA sequencing. Sequencing quality was good with no sample discarded and we identified 1664 ASVs in total. As is shown in **Figure 1A**, the alpha diversity was not significantly different among the three groups, indicating the similarity of global composition between NP and non-NP patients. The species richness rarefaction curves (**Figure 1B**) flattened out gradually, indicating that the number of individual samples was reasonable. The Venn diagram (**Figure 1C**) showed the common and unique ASVs detected in NP, non-NP, and HC groups, respectively. As displayed in CPCoA of weighted UniFrac distances (**Figure 1D**), a significant difference in beta diversity was detected in the microbial compositions among the three groups (*p* = 0.001).

**Figure 2:**
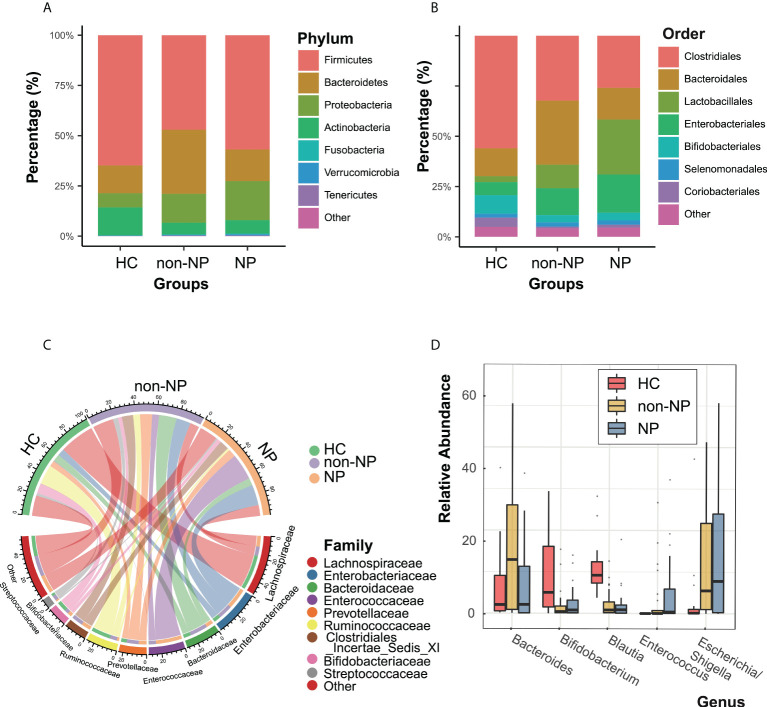
Taxonomic features of NP, non-NP and healthy control groups. Relative abundances of bacteria between groups at the phylum **(A)**, order **(B)**, family **(C)** and genus **(D)** levels. NP, necrotizing pancreatitis; non-NP, non-necrotizing pancreatitis.

Taxonomic profiles at different levels of the three groups were presented in **Figure 2**. *Firmicutes*, *Actinobacteria*, *Bacteroidetes*, and *Proteobacteria* were the predominant phylum in the three groups (**Figure 2A**). At the order level (**Figure 2B**), the NP and the non-NP group showed lower relative abundances of *Bifidobacteriale* and *Clostridiales* and higher relative abundance of *Lactobacillales* compared with the HC group. In the NP group, *Clostridiales* and *Bacteroidales* were less abundant, while *Lactobacillales* and *Enterobacteriales* were more abundant, comparing with the non-NP group. As for family level, a chore diagram was constructed to visualize the connections between microbiota composition and the three groups (**Figure 2C**). Higher abundance of *Enterococcaceae* and lower abundance of *Bacteroidaceae* in the NP group were depicted comparing with the non-NP group. At the genus level, as shown in **Figure 2D**, NP exhibited a lower relative abundance of *Bifidobacterium* and *Blautia*, which were considered to be probiotic candidates. We also noticed an increased relative abundance of *Enterococcus* and *Escheichia/Shigella* comparing with non-NP. Overall, our finding suggested that AP (both non-NP and NP) might be associated with the decrease of certain taxa of probiotic microbes and the increase of opportunistic pathogens as well.

### Specific taxonomic signatures in NP patients

**Figure 3:**
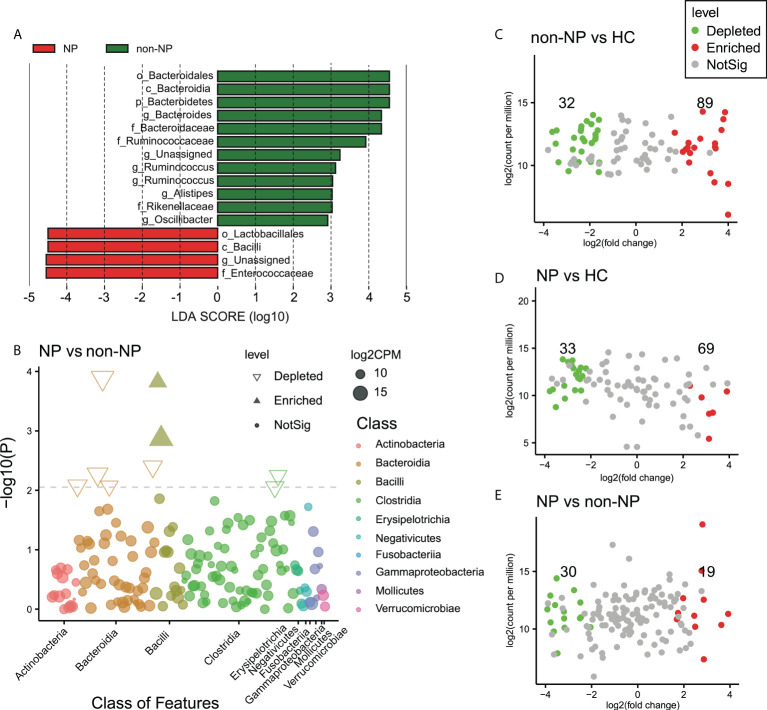
Differential bacteria taxa and ASVs between the NP, non-NP and HC groups **(A)** LefSE analysis comparison between the NP and the non-NP groups, bacterial taxa are listed with a LDA score > 2.0. Microbial taxa enriched in non-NP group were highlighted in green; c, o, f, and g represent class, order, family and genus, respectively. **(B)** Manhattan plot of differential taxa between NP and non-NP groups. The x axis represents the microbial taxa at the class level ranked by alphabetical order, and y axis represents -log10 (p value). Filled triangles, hollow inverted triangles, and solid circles indicate ASVs enriched, depleted, and without significant difference, respectively. The color of each marker represents the different taxonomic affiliation of the ASVs, and the size corresponds to their relative abundances using log2 transformed CPM values. **(C–E)** Volcano plot. Each point represents an ASV, and significantly different ASVs are colored (non-NP vs HC, NP vs HC, NP vs non-NP; green = depleted in the former group; red = enriched in the former group; gray = not significant). NP, necrotizing pancreatitis; non-NP, non-necrotizing pancreatitis; ASV, amplicon sequence variants.

A LefSE analysis was performed to reveal the predominant bacterial taxa in NP and non-NP groups (**Figure 3A**). Manhattan plot (**Figure 3B**) delineated differential taxa between the NP and the non-NP group at the class level, in which *Bacilli* was enriched, while *Bacteroidia* and *Clostridia* were depleted in the NP group. The volcano plots based on edgeR analysis, as shown in **Figures 3C–E**, depicted that there were 121, 99, 49 ASVs with discrepant read counts statistically different between non-NP and HC group, NP and HC group, NP and non-NP group, respectively. 32 ASVs were depleted and 89 ASVs were enriched in non-NP as compared with HC, 33 ASVs were depleted and 69 ASVs were enriched in NP group as compared with HC, 30 ASVs were depleted and 19 ASVs were enriched in NP group as compared with non-NP.

### Gut microbiota composition was correlated with clinical indicators

**Figure 4:**
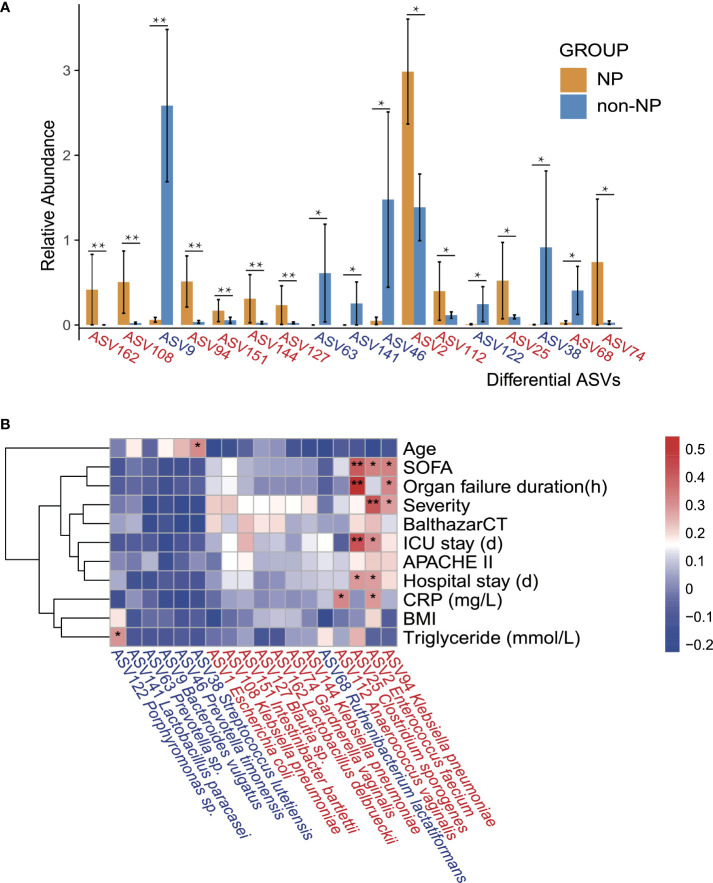
Differential ASVs between NP and non-NP groups and the correlation with clinical indicators. **(A)** Bar plot showing the relative abundance of differential ASVs between the NP and the non-NP groups. Values represent mean ± SEM, SEM-standard error of mean. *p < 0.05, **p < 0.01, edgeR test. **(B)** Spearman correlations between differential ASVs and clinical outcomes as well as indicators of disease severity. ASV, amplicon sequence variants; SOFA: the Sequential Organ Failure Assessment score; Severity: MAP-mild acute pancreatitis, MSAP-moderately severe acute pancreatitis and SAP-severe acute pancreatitis; BalthazarCT: Balthazar score within the CT severity index for grading of acute pancreatitis; APACHE II: the Acute Physiology and Chronic Health Evaluation II score; CRP: C-reactive protein; BMI: body mass index; Positive (red) or negative (blue) correlation are shown by two-color heatmap, with asterisks denoting statistical significance (*p < 0.05, **p < 0.01). ASVs enriched in the NP group were highlighted in red, and ASVs enriched in the non-NP group were in blue.

Spearman correlation analysis was performed to recognize the connection between gut microflora and clinical indicators involving demographic characteristics, laboratory examinations, severity, and outcomes. *Enterococcus faecium* (ASV2), which belongs to the family *Enterococcaceae*, had a higher abundance in NP compared with non-NP (**Figure 4A**), bearing moderate to strong positive correlation with SOFA, disease severity, ICU stay, hospital stay, and CRP level. Likewise, *Clostridium sporogenes* (ASV25), found more abundant in the NP group, had a strong and positive correlation to SOFA, organ failure duration, and ICU stay while moderately positively correlated to hospital stay. *Klebsiella pneumoniae* (ASV94), within the order of *Enterobacterales*, had a moderate positive correlation with SOFA, organ failure duration, and disease severity.

### Altered functional pathways in microbiota of NP patients

**Figure 5:**
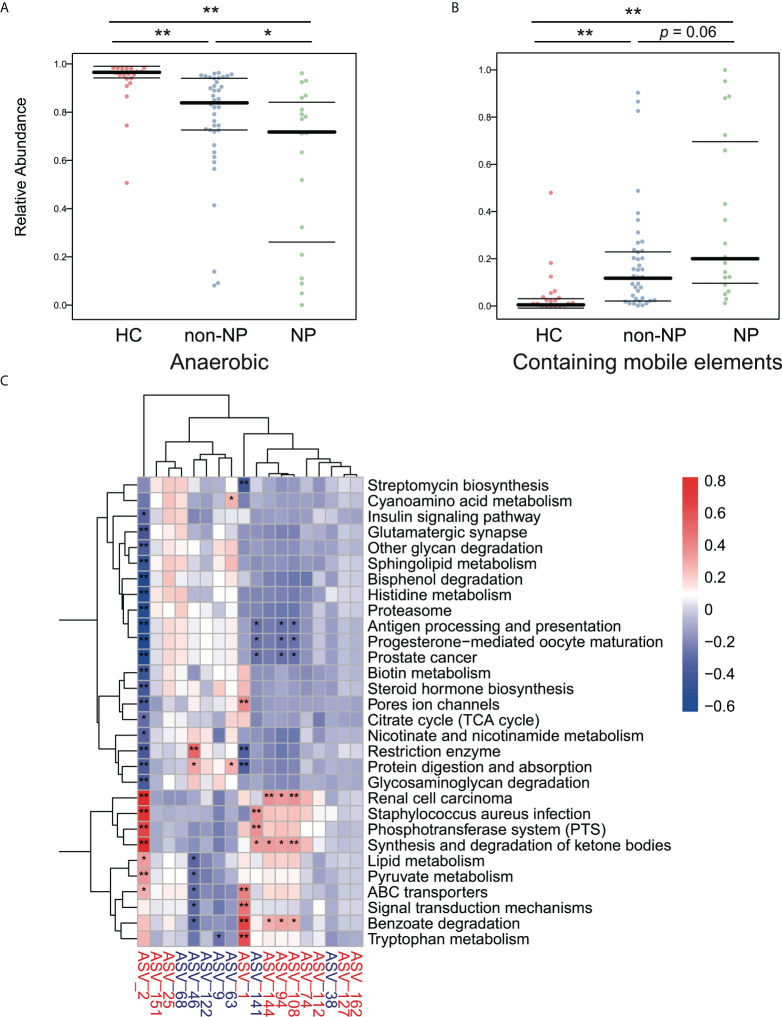
Predicted phenotypes of intestinal microflora in the NP, non-NP and HC groups. **(A, B)** Predicted relative abundance of anaerobic bacteria and bacteria containing mobile elements based on the BugBase database. *p < 0.01, *p <0.05, ns, not significant, by pairwise Mann-Whitney-Wilcoxon tests. **(C)** Spearman correlations were calculated between pathways predicted by PICRUSt2 that differed between the the NP and the non-NP groups and differential ASVs. Rows: Differential pathways predicted by PICRUSt2 between the NP and NNP groups; Columns: Differential ASVs. ASVs highlighted in red were enriched in the NP group, and ASVs highlighted in blue were depleted. *p < 0.05, **p < 0.01.

We performed a BugBase analysis to predict the bacterial metabolic phenotypes in NP, non-NP, and HC groups, and the anaerobes abundance and mobile elements possession predictions are shown in **Figures 5A, B**. Compared to HC, non-NP had a significantly lower relative abundance of anaerobes (*p* < 0.01), while NP had a much lower relative anaerobes abundance in contrast to non-NP (*p* < 0.05). In terms of bacteria containing mobile elements, a significantly higher abundance was detected in the non-NP group as compared to the HC group (*p* < 0.01), while there was no significant difference between NP and non-NP (*p* = 0.06). Moreover, we analyzed the metabolic pathways of gut microflora based on PICRUSt2 (**Figure 5C**). We found that *Enterococcus faecium* (ASV2), *Lactobacillus paracasei* (ASV141), *Klebsiella pneumoniae* (ASV94, ASV108, and ASV144), and *Escherichia coli* (ASV1), had positive correlation with microbial gene functions concerning staphylococcus aureus infection, phosphotransferase system, ABC transporters, synthesis and degradation of ketone bodies, while they had a negative correlation with antigen processing and presentation, protein digestion and absorption, and restriction enzyme. While for *Prevotella timonensis* (ASV46), positive correlation to microbial gene functions related to restriction enzyme, protein digestion and absorption was observed. They had negative correlation with benzoate degradation.

**Figure 6:**
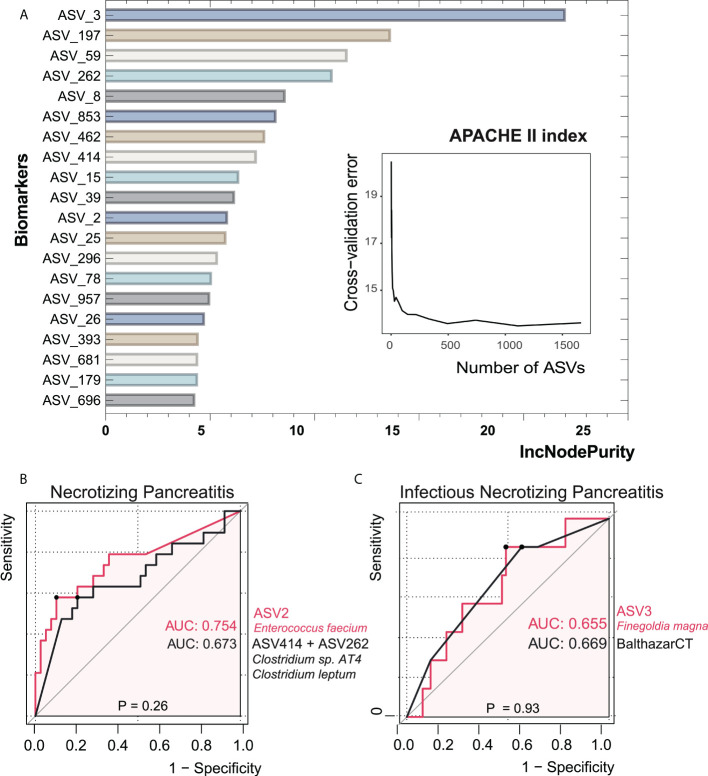
Bacterial taxonomic biomarkers of the APACHE II index in acute pancreatitis. **(A)** The top 20 biomarker bacterial classes were identified by applying random forests regression to their relative abundance values. Potential biomarker taxa are ranked in descending order of importance to the accuracy of the model. The inserted figure shows the 10-fold cross-validation error as a function of the number of ASVs used to regress against the APACHE II index in order of variable importance. **(B, C)** ROC curves showed the ability of specific microbiome biomarker in discriminating necrotizing pancreatitis and infectious necrotizing pancreatitis. AUC, area under ROC curve.

### Potential gut microbial features were predictive of NP severity

In order to investigate the value of gut microbial signatures in forecasting NP severity, we applied random forest to identify taxa associated with poor outcomes, as shown in **Figure 6**. *Finegoldia magna* (ASV3) showed the maximal prediction capacity as a biomarker of NP when performing regression analyses with the APACHE II index. *Enterococcus faecium* (ASV2) had the highest AUC of 0.754 among all single ASVs in the setting of distinguishing NP and non-NP. The combination of two *Clostridium* species (ASV414+ASV262), which had a relatively low abundance in NP groups compared with non-NP, had comparable AUC value with ASV2. We then asked whether the composition of gut bacteria was predictive of infected NP. It turned out that there was not significant difference in AUC between ASV3 as a biomarker and Balthazar CT under the circumstance of infected NP (*p* = 0.93), suggesting the considerable predictive potential of ASV3.

## Discussion

We explored the intestinal microbiota in patients with NP, non-NP, and healthy controls. Our analysis demonstrated a significant difference in microbiota composition among the three groups. We then proposed that bacterial biomarkers such as diversity, relative abundance feature, and specific ASVs might be useful to recognize patients who are likely to develop NP.

In our study, CRP, APACHE-II, and SOFA score differ significantly in NP and non-NP, which may be attributed to the severity of NP compared with non-NP. Many researchers have investigated a series of scoring systems from AP severity prediction. Prospective studies compared Ranson score, APACHE II, CTSI, BISAP score, and SOFA score, which were commonly used in clinical settings, in predicting necrosis development (32–34). CTSI had the highest positive predictive value compared with Ranson score, APACHE II and BISAP (32). However, CTSI is limited by accessibility to contrast enhanced CT (CECT) in the early stage of AP. BISAP score and APACHE II score did not have comparable predicting potential for NP (32). Besides, Ranson score consists of parameters assessed at both admission and after 48h, which is to the disadvantage of early clinical judgement. SOFA score, which was not specifically designed for AP, was more closely related to systemic complications and mortality, rather than local complications such as NP (8, 34). Other researchers generated *de novo* single factor or combination parameters for NP prediction. For instance, CRP (35), red cell distribution width (10) and immature granulocyte percentage (11) were also suggested as predictors of NP. However, these clinical laboratory items are likely to fail considering they are not specific for the complex pathophysiology of NP. A research team recently used machine learning strategy to process data from 2387 AP patients measured in the first 24h of clinical admission (36). They released an on-admission prediction model for NP online which stressed the impacts of gender, total white blood cell count, glucose, alkaline phosphatase, and CRP. However, more clinically relevant data should be included to improve the model. Also, model prediction efficacy usually shifted with various cohort (37).

As for infected NP prediction, studies suggested that sustained increase of procalcitonin (PCT) as a predictor for infected PN if repeatedly measured for a 2-week period (38). Nevertheless, PCT may be false positive in AP patients due to endotoxin translocation from intestine without infection (39). Inflammatory cytokines, such as interleukin 6 (IL6), are also predictor candidates, nevertheless they may be more difficult to access compared with PCT (40). One study reported that the peak of hematocrit (HCT), blood urea nitrogen (BUN), PCT, and CRP within 48h of admission were independent factors for infected PN, and parameters combination added to prediction accuracy. However, the limited sample size and the retrospective design raised issues of potential bias and questionable generalizability. Also, they diagnosed infected PN based on culture confirmation solely, which may missed false-negative cases.

Intestinal barrier injury, microbial translocation, and local inflammation have been proved to have an essential role in the generation and escalation of AP (13, 41), in which dysbiosis of the gut microbiota is a major contributing factor to intestinal damage (42). A previous study conducted by *Zhu et al.* showed that AP significantly triggered dysbiosis, and that gut microbiota dysbiosis increased the severity of AP in both animal studies and clinical settings (43). Besides, our previous studies also unveiled varying compositions and functions of gut microbiota in mild and non-mild AP patients, suggesting that altered microbiota might be responsible for the progression of AP (16). Although specific mechanism by which intestinal microflora affects escalation of NP remains unclear, some fundamental progress has been made recently. *Zheng et al.* reported that certain gut microbiome, for instance, non-pathogenic commensal *Escherichia coli*, had harmful potential in the human intestine and could aggravate NP through targeting intestinal epithelia (44).

We found that the microbial diversity in NP, species richness in particular, was lower than that in non-NP. Of note, the two groups had different microbiota composition. It is commonly acknowledged that the intestinal microbiota shapes a sophisticated community that bears various physiological and immune-modulating capacities (45). They also play a pivotal role in stimuli for antimicrobial peptides induction (46). Besides, the shift in the balance of symbionts and pathobionts (45), or dysbiosis, has been evidenced to be associated with ulcerative colitis and Crohn’s disease (47). Therefore, we presume that the decreased diversity may result in reduced ecological viability and dysbiosis in the intestine, implicating a weaker defense against pathogenic microorganisms, rendering the gut vulnerable to bacterial translocation that is essential for generating systemic inflammatory response, organ dysfunction, and local complications in AP.

There was consistency of relative abundance throughout different taxonomic levels. Opportunistic pathogens like *Enterococcus* were more abundant, while probiotic microbes like *Bacteroides* were less abundant in NP comparing with non-NP. Certain *Bacteroides* species, for instance *Bacteroides xylanisolvens*, *Bacteroides ovatus*, and *Bacteroides uniformis*, played a pivotal role in boosting the gut IgA production, which restored intestinal environment stability by restricting the microorganisms and endotoxin from invading gut epithelial cells, modulating microorganism colonization, and facilitating bacterial clearance (48–50).

*Enterococci*, a common inhabitant of human gastrointestinal tract, usually affect debilitated patients with prolonged hospital admission (51). Our previous study demonstrated that *Escherichia/Shigella* and *Enterococcus* were linked to poor prognosis of AP patients, and were commonly culprit pathogens of infected pancreatic necrosis (21). An increased abundance of *Enterococcus* may lead to higher disease severity (14, 16). Of note, infected NP were recommended to be treated with antibiotics in early disease stage (52). However, certain empiric antibiotics used in hospitalized patients might reduce abundance of the Gram-negative organisms in the GI tract. Depletion of the Gram-negative microbiota weakens the antimicrobial activity towards Gram-positive bacteria including enterococci, leading to overgrowth of enterococci (53, 54).

Besides, in the process of developing antibiotic resistance, certain enterococci lineages, *Enterococcus faecium* for instance, can colonize the intestine and disseminate to cause systemic infection (51). In this study, *Enterococcus faecium* was found enriched in NP compared with non-NP. Further, *Enterococcus faecium* (ASV2) performed best in discriminating NP and non-NP patients based on AUC. A previous study showed that *Enterococcus faecium* was one of the most frequently identified microbes in patients with infected pancreatic necrosis (55). Simultaneously, we found that *Enterococcus faecium* was not only correlated with clinical indicators such as CRP, SOFA, disease severity, ICU stay, and hospital stay, but was also connected with many microbial gene functions. Therefore, further studies on the mechanisms by which *Enterococcus faecium* boosters NP are required. In addition, NP patients had a much lower relative anaerobes abundance than non-NP patients, giving a hint of the selection of antibiotics when dealing with NP.

To be noticed, a combination of clostridium species had similar performance in discriminating NP and non-NP compared with *Enterococcus faecium.* Previous studies revealed protective effect of certain clostridium species in AP by regulating pancreatic-gut homeostasis. Supplemented *Clostridium butyricum strains* to mice model protected against AP (56). At the pancreas level, *Clostridium butyricum* inhibited dendritic cell and neutrophil infiltration, inflammasome activation, and certain pro-inflammatory pathways. At the gut level, *Clostridium butyricum* attenuated barrier dysfunction and intestinal inflammation, therefore prevented *Enterococcus* and *Escherichia coli* from penetration into pancreas. For inflammatory bowel disease (IBD) patients, significant reduction of *Clostridium leptum* was observed, which contributed to reduced short chain fatty acids and dysbiosis (57). Analysis of gut microbiota in AP patients with infection complication also revealed decreased bifidobacterial such as *Clostridium leptum (* 58). Probiotic supplementation worth further investigation in AP patients.

Our random Forests regression analysis indicated that *Finegoldia magna* (ASV3) was the most robust microbial biomarker to predict the prognosis compared to the APACHE II score. Furthermore, we found that in the setting of infected NP, AUCs of *Finegoldia magna* and Balthazar CT were comparable, implicating that *Finegoldia magna* could be a potential substitute to assess disease severity of infected NP. *Finegoldia magna*, a commensal Gram-positive anaerobic coccus (GPAC) that colonizes gastrointestinal tract, is an important opportunistic pathogen. However, due to challenges of high-quality anaerobic specimens collection and difficulties in cultivation, its true impact in clinical settings is possibly underestimated (59).. *Finegoldia magna* and its soluble proteins *Finegoldia magna* adhesion factor (FAF) and superantigen protein L activate human neutrophils, promote inflammatory response, and hinder antibacterial peptides or proteins, which may increase virulence (60). Biofilm formation hampers successful antibiotic therapy and results in chronic infections. Relative high resistance of clindamycin (in some studies more than 30.0%) or quinolone (often more than 30%) was observed. And though quite rare, studies also observed resistance of metronidazole, penicillin G, and chloramphenicol (61). 16S rRNA PCR increases the possibility of *Finegoldia magna* detection compared to traditional methods, thus conducive to antibiotic selection and NP outcome prediction. *Finegoldia magna* has virulent factors such as subtilisin-like extracellular serine protease (SufA), superantigen protein L, and *F. magna* adhesion factor (FAF) that prevent clearance by innate defense system. Profound penetration and dissemination of the infections through skin are realized by capsule and tissue- destroying enzymes, as well as SufA. Biofilm contributes to chronic infection (61). However, the relationships between *Finegoldia magna* and the pathophysiology of NP or infected NP remain to be investigated.

In clinical setting, many microorganisms and their constituents have been transformed into robust diagnostic tests for certain diseases. For instance, *Clostridium difficile* and *C. difficile* Toxin A & B (CDAB) are practical diagnostic tests for antibiotic-associated colitis. Therefore, *Enterococcus faecium*, *Finegoldia magna*, and their virulence factors are promising to become cost-effective diagnostic markers.

As for function prediction analysis, we found that species more abundant in NP patients were correlated positively with microbial gene functions related to synthesis and degradation of ketone bodies, and benzoate degradation, while they had a negative correlation with antigen processing and presentation, protein digestion and absorption. While for species less abundant in NP patients, positive correlation to microbial gene functions related to restriction enzyme, protein digestion and absorption was observed, as well as negative correlation with benzoate degradation. A study observed that β-hydroxybutyrate (βOHB), a type of ketone body in circulation, was elevated in patient with non-SAP, but not in SAP (62). Then the researchers used mild or severe form of AP mice to demonstrate that βOHB weakened the pancreatic and systemic proinflammatory macrophages activation by means of class I histone deacetylases. Apart from endogenous ketogenesis, gut microbiota may affect the synthesis and degradation of ketone bodies, which may trigger NP, a non-mild form of AP. Activation of endogenous ketogenesis or supplementation of exogenous βOHB may help with AP prevention and treatment. Intestinal microbial growth may release metabolism excreted by urine. Urine benzoate was elevated in intestinal bacterial overgrowth patients, and was considered a dysbiosis marker (63). Benzoate degradation pathway connected host adrenergic stress to strengthened microorganism virulence, and was implicated in IBD (64). NP and non-NP groups possibly had different benzoate degradation metabolic phenotype, which might imply dysbiosis and bacterial virulence in terms of mechanism. Possible preventive treatment measures regarding metabolic products are worth trying.

Our pilot study had several limitations. Firstly, we took gut microbiome samples at the single time point of admission, but longitudinal monitoring at different time points would enable better understanding of dynamic changes in AP. Secondly, the sample size was limited, thus a multicenter study is needed to verify our results. Thirdly, microbiome taken from various segments of the intestine, whole genome sequencing, and multi-omics will hopefully provide multilevel information to better interpret the mechanisms.

Our study indicated that patients with NP had altered gut microbiome which was predictive for NP patients with worse outcome. *Enterococcus faecium* and *Finegoldia magna*, microbial biomarkers for NP and infected NP, warrant further investigation of their mechanism and clinical application. Our findings may help early prediction of disease progression and inform clinical decision-making in patients with AP.

## Conclusion

In conclusion, NP patients had distinct features of gut microbiota which can be used to predict disease severity. These features were related to poor clinical prognosis. *Enterococcus faecium* and *Finegoldia magna* were potential microbial biomarkers for prediction of NP and infected NP. Further multi-omics, animal studies, and human trials are required to validate our findings and demonstrate the underlying mechanisms.

## Data availability statement

The datasets for this article are not publicly available due to concerns regarding participants/patient anonymity. Request to access the datasets should be directed to the corresponding author.

## Ethics statement

The studies involving human participants were reviewed and approved by PUMCH Ethics Committee (Identifier: JS1826, Date of approval: 20th February 2018. Period of validity: February 2018 to August 2020). The patients/participants provided their written informed consent to participate in this study.

## Author contributions

Conceptualization, MZ, XH, DW. Methodology, XH and DW. Software, XH and YF. Validation, MZ, XH and DW. Formal Analysis, XH, YF. Investigation, MZ, LG, ZH, and LJ. Resources, DW. Data Curation, XH and DW. Writing—Original Draft Preparation, ZY (Zihan Yang) and YF. Writing—Review & Editing, MZ, XH, and DW. Visualization, XH and YF. Supervision, XH, DW. Project Administration, XH, DW. Funding Acquisition, DW. All authors have read and agreed to the published version of the manuscript.

## Funding

This study was funded by grants from Chinese Natural Science Foundation, grant number 32170788, Beijing Natural Science Foundation, grant number 7202152, and National High Level Hospital Clinical Research Funding, grant number 2022-PUMCH-A-026

## Conflict of interest

The authors declare that the research was conducted without any commercial or financial relationships that could be construed as a potential conflict of interest.

## Publisher’s note

All claims expressed in this article are solely those of the authors and do not necessarily represent those of their affiliated organizations, or those of the publisher, the editors and the reviewers. Any product that may be evaluated in this article, or claim that may be made by its manufacturer, is not guaranteed or endorsed by the publisher.
