# Supplementary figures and images for: Gut microbiota on admission as predictive biomarker for acute necrotizing pancreatitis
Source: Front Immunol. 2022 Aug 29;13:988326. doi: 10.3389/fimmu.2022.988326 (PMC9466706; doi:10.3389/fimmu.2022.988326)

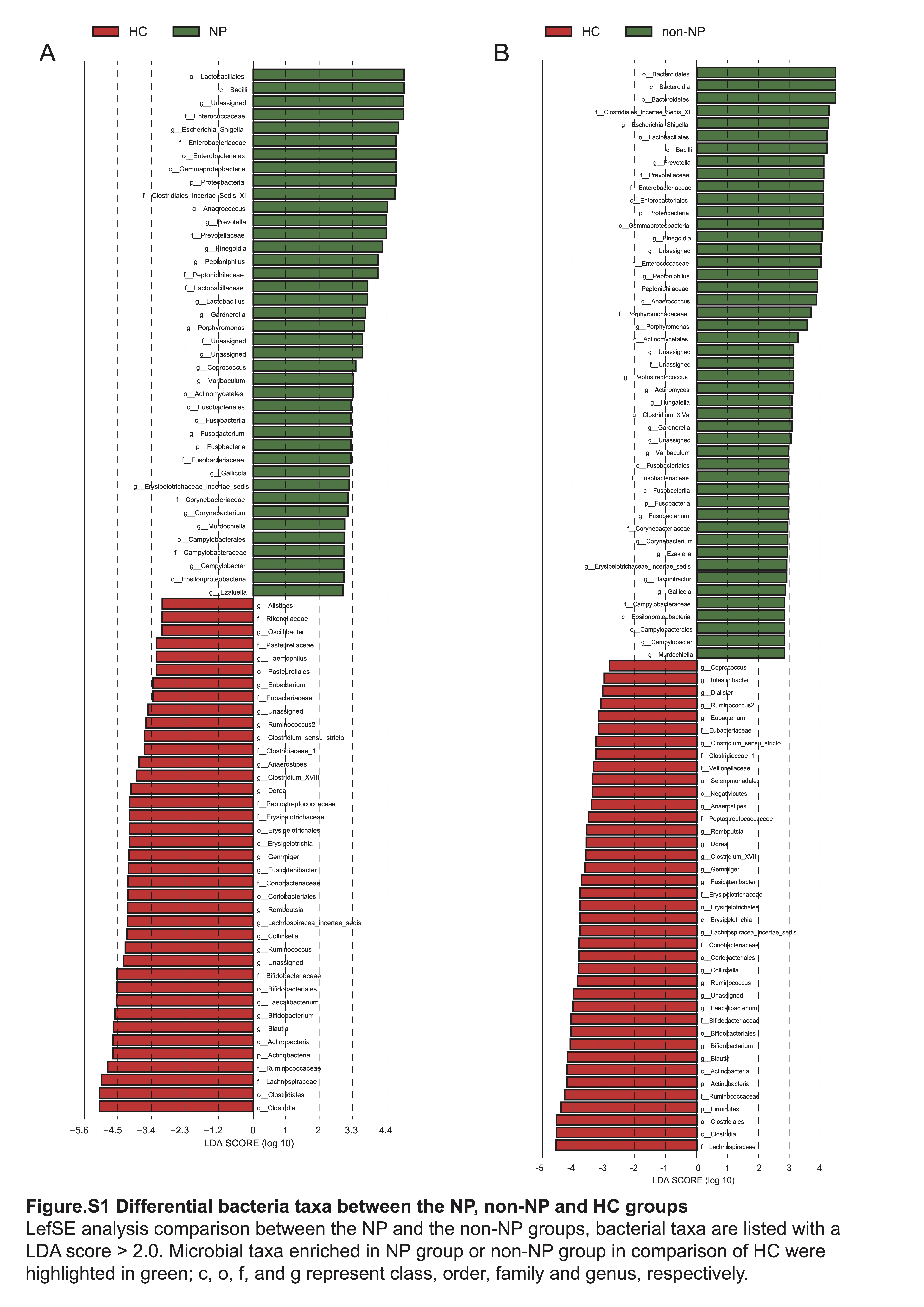

Supplement: Supplementary file 2 [file Image_1.jpeg]
